# Nonregional small fibre neuropathy in cases of autoimmune autonomic neuropathy

**DOI:** 10.1007/s00415-022-11340-3

**Published:** 2022-09-09

**Authors:** Andrea Maier, Romina Kapfenberger, Istvan Katona, Joachim Weis, Jörg B. Schulz, Roman Rolke

**Affiliations:** 1 Department of Neurology, Medical Faculty RWTH Aachen University, Aachen, Germany; 2 Institute of Neuropathology, Medical Faculty, RWTH Aachen University, Aachen, Germany; 3 JARA-BRAIN Institute Molecular Neuroscience and Neuroimaging, Juelich Research Center GmbH and RWTH Aachen University, Aachen, Germany; 4 Department of Palliative Medicine, Medical Faculty, RWTH Aachen University, Aachen, Germany

**Keywords:** Small fibre neuropathy, Autoimmune autonomic neuropathy, Skin biopsy, Quantitative sensory testing

## Abstract

**Objective:**

Autonomic small fibre neuropathy is described in patients with autoimmune autonomic neuropathy (AAN). Few data are available on somatosensory function and skin biopsies in AAN.

**Methods:**

Retrospective analysis of 17 patients (51.2 ± 6.8 years, *n* = 7 males) with AAN, including autoantibodies, quantitative sensory testing (QST, *n* = 13) and intraepithelial nerve fibre density (IENFD) in skin biopsy (*n* = 16). QST was performed according to the DFNS protocol over hands and feet dorsum. QST data were compared to healthy controls. Comparison of antibody-positive and antibody-negative cases.

**Results:**

70.6% of patients were antibody positive. 82.4% described at least one episode with sensory symptoms. Skin biopsies revealed reduced IENFD in 58.8% of patients, whereas neuropathic pain was only present in 41.2%. QST showed a nonregional increase for nonpainful thermal and mechanical detection rather than for mechanical pain thresholds. Compared to healthy controls, sensory loss for cold and warm detection thresholds and for the thermal sensory limen—the temperature difference between alternating warm and cold stimuli—was found on hands and feet (all *p* < 0.05). For nonpainful mechanical stimuli, the vibration detection threshold on the hand was increased (*p* < 0.05). Of all pain thresholds, only the mechanical pain threshold was elevated for pinprick stimuli to the feet (*p* < 0.05).

**Interpretation:**

Findings are consistent with a sensory small fibre more than large fibre neuropathy in AAN. Sensory loss was comparably distributed across hands and feet, indicating that nerve fibre dysfunction was rather generalized. Serostatus was not a significant predictor of the small fibre deficit present in AAN.

## Introduction

Autoimmune autonomic neuropathy (AAN) is a rare autonomic neuropathy that mainly affects middle-aged patients [1, 2]. Several autoantibodies are suspected to mediate AAN, and some authors classify the disease into antibody-positive and antibody-negative cases [1, 3], with elevated ganglionic acetylcholine receptor antibodies (gACHR-Ab) in 50% of patients [1]. N-type calcium channel antibodies [4] have also been described. Sympathetic dysfunction is seen predominantly in seronegative patients according to Golden et al. [3], while other authors do not describe any significant differences between seropositive and seronegative patients [1]. Autonomic nerve fibres are small, thin myelinated and unmyelinated nerve fibres [5]. Some AAN patients describe neuropathic pain [3], similar to that in patients with typical small fibre neuropathy (SFN). In SFN, A-delta and C fibres that are involved in pain and temperature sensation are affected [5]. Symptoms of SFN may also include autonomic complaints, e.g., hypohydrosis [5]. Although autonomic dysfunction is common in SFN, sparse data are available on the frequency and distribution of small fibre damage in patients with AAN. Thus, this retrospective study was designed to investigate small fibre function in AAN patients regarding the frequency, distribution and characteristics of sensory deficits, analysing data from non-invasive quantitative sensory testing (QST) and skin biopsies performed in routine clinical examinations. Moreover, the study aimed to disclose a potential difference between antibody-positive and antibody-negative patients regarding sensory deficits and IENFD.

## Subjects/materials and methods

Data from patients with a clinical diagnosis of an AAN who were diagnosed and treated in our outpatient clinic for autonomic disorders between July 2012 and June 2019 were retrospectively evaluated. Inclusion criteria for the AAN group were based on current literature concerning the occurrence, course, symptoms and diagnosis of AAN. Patients were assigned to the diagnosis of AAN if the following criteria were fulfilled:

1. Patients experiencing initial symptoms between the ages of 35 and 60 years, oriented at the mean values of symptom occurrence by Nakane et al. [1] and Suarez et al. [2] Typical causes and course of AAN: autoimmune or neoplastic diseases [1] and/or a typical clinical course (at least 1): previous event:, e.g., viral infection [1, 2], onset: subacute or gradual [1], orthostatic hypotension as the first autonomic symptom [1, 3] and/or a monophasic course without complete recovery [2]
2. Typical clinical presentation (at least 5 typical autonomic symptoms or at least 3 autonomic symptoms combined with 1 extra-autonomic symptom)
  Autonomic complaints [1, 2]: hypo/anhidrosis/heat intolerance, pupillary dysfunction, circulatory disorder, bladder disorder, sicca syndrome, dry skin, gastrointestinal symptoms and sexual dysfunction.
  Extra-autonomic symptoms: sensory dysfunction, especially dysesthesia and neuropathic pain [2, 3] and central nervous system involvement –, e.g., psychiatric symptoms [1].
  Typical examination results (at least 2): pathological skin biopsy [6], elevated gACHR-Ab [1] or N-type calcium channel antibodies [4], cardiovagal and adrenergic dysfunction on tilt table testing [2], low norepinephrine levels when supine [1], sudomotor dysfunction [2] in the sympathetic skin response and/or pinprick disease [3]—abnormalities in mechanical testing (QST).

The following exclusion criteria were defined to rule out Lambert Eaton syndrome: at least one typical examination result (typical electrophysiology or detection of PQ—calcium channel antibodies [7]) and one typical clinical sign (proximal muscle weakness, abnormal cranial nerves/oculobulbar symptoms and areflexia [8]) or two typical examination results.

Patients were excluded if pure autonomic failure (PAF) was suspected. PAF had to be considered if there were at least two clinical signs: olfactory complaints [9], advanced average age of more than 60 years [9], injuries of themselves or others while sleeping [9], hyperreflexia or Babinski’s sign [10].

Patients were classified as seropositive if they had antibodies against gACHR [1], N-type calcium channel [4] or other antibodies associated with autoimmune diseases affecting the autonomic nervous system [against gangliosides, potassium channel and glutamate-decarboxylase-65 (GAD-65)].

In the clinical routine, each patient was examined by an experienced physician as follows: detailed history, physical examination, blood sampling, QST, skin biopsy and tilt table testing with continuous blood pressure measurement.

The patient’s history included the experienced symptoms, the course of the disease, secondary diagnoses and demographic data such as age and sex. For analysis, the anamnestic data were processed in a qualitative manner and classified into subgroups based on complaints of the eyes, gastrointestinal tract, sweating, mouth and skin, bladder function, circulatory function, and neuropathic pain. Relevant subgroups for secondary diagnoses were other autoimmune diseases, neoplasia, Ehlers–Danlos-syndromes, systemic lupus erythematosus, Sjögren’s syndrome, and stiff person syndrome. Further criteria were the first autonomic symptom and the date of first diagnosis.

The physical examination included sensitivity, cranial nerves, pupillomotor function, temperature sensation and reflexes, as well as skin condition. In clinical routine, the blood sample (serum gel tube) taken was tested for antibodies against the following structures: N-type calcium channel (Radioreceptor assay, RRA), g-ACHR, (RRA), gangliosides (ELISA), GAD-65 (enzyme immunoassay), PQ-type calcium channel (RRA) and potassium channel (radioimmunoassay). These antibody samples were analysed in an external laboratory (SYNLAB, Leverkusen, Germany).

QST was used to examine small fibre function. It was performed in all patients under standardized conditions as described previously [11] on the dorsum of one hand and foot (mostly the left).

Skin biopsies were obtained from the right lateral lower leg (10 cm above the lateral malleolus) under local anaesthesia with 0.5 ml prilocaine and epinephrine (Xylonest, Aspen Germany GmbH, Munich, Germany). Intraepithelial nerve fibre density (PGP9.5 immunofluorescence), axon branching and focal swellings (> 1.5 µm), rarefication of the subepidermal plexus and innervation of imaged sweat glands were examined as described previously [12]. The IENFD was considered reduced when below the published normative values for the respective sex and age group [13]. Differences of autonomic symptom burden were compared between AAN patients with and without nerve fibre damage in the skin biopsies concerning autonomic symptoms, norepinephrine levels and tilt table diagnosis.

Two out of the following three criteria must be fulfilled for the diagnosis of a definite SFN [5]: (1) At least two clinical signs of SFN (e.g., reduced temperature sensitivity, or pinprick hypoalgesia, allodynia, hyperalgesia), (2) abnormal thresholds in QST and/or (3) reduced intraepidermal nerve fibre density (IENFD) in a punch biopsy taken from the distal leg.

### Statistics

IBM SPSS version 25/27, STATISTICA 7.1 (Statsoft, Inc.) and Excel for Mac were used for statistical analysis. Qualitative results of anamnesis and physical examination were evaluated by frequency counts. The chi square test was used to investigate possible differences between seropositive and seronegative patients and in AAN patients with and without small fibre nerve damage regarding nominal values, and the Mann–Whitney *U* test was used to assess metric values.

QST values were compared with healthy controls (age: 55.0 ± 5.7 years; mean ± SD) that were matched for age and sex derived from the internal clinical database. After log- and z-transform, QST data were analysed using a two-way mixed ANOVA comparing groups (AAN vs. controls as ‘between factor’) and body regions (hand vs. foot as ‘within factor’). Differences in QST values depending on the serostatus were investigated with multiple ANOVAs based on *z* score QST data. Differences in IENFD were assessed using a Mann–Whitney *U* test.

Correlations of IENFD and QST values were assessed using correlation coefficients with IENFD as the determining factor. Missing data were removed pairwise. Finally, the symptomatic pattern, the presence of antibodies and the results of QST and skin biopsies of AAN patients were also presented descriptively.

## Results

**Fig. 1:**
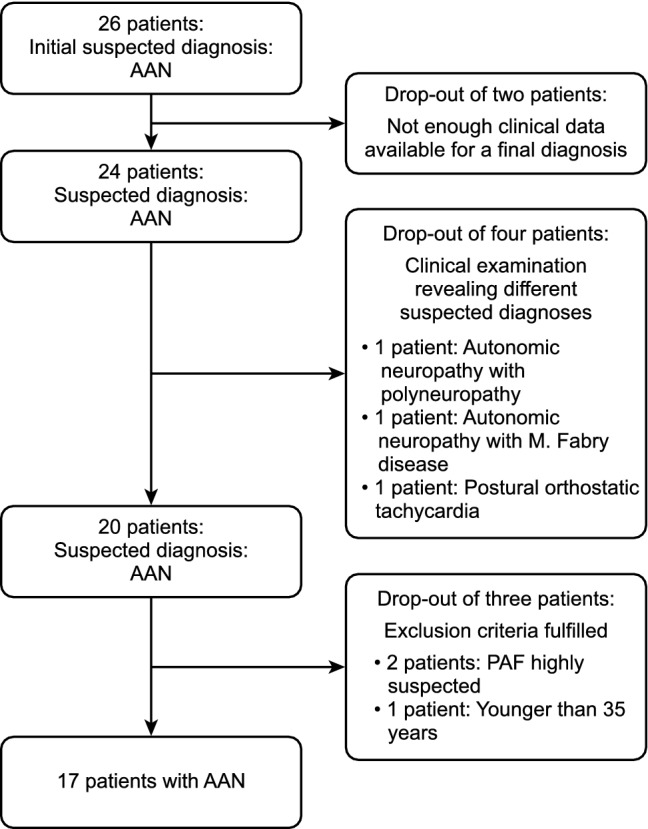
Flowchart of the patient population based on the inclusion and exclusion criteria. This figure was created using Word

Data from 26 patients (see Fig. 1) with a clinical diagnosis of an AAN were retrospectively evaluated. Of these, 17 patients (age: 51.2 ± 6.8 years, *n* = 7 male) fulfilled all inclusion criteria. More women (58.8%) than men (41.2%) had a diagnosis of an AAN. A total of 70.6% (*n* = 12) of the patients (50.5 ± 7.6 years, 7 females, 5 males) were seropositive, most frequently (47.1%) with antibodies against N-type calcium channels (*n* = 8/17, 52.25 ± 7.7 years, 4 females). gACHR-Ab was elevated in 17.6% (*n* = 3/17, 2 females) (Table 1). Antibodies against GAD-65 were found in 23.5% (*n* = 4/17, 3 females), against the voltage-gated potassium channel in 11.8% (*n* = 2/17), and against gangliosides in 5.9% (*n* = 1/17). 58.8% (10 patients) had a known underlying autoimmune disease, the course of the disease was monophasic without complete recovery in all patients. An acute onset was only reported by one patient with a previous toxoplasmosis infection. Orthostatic hypotension occurred as the first autonomic symptom in 43.8% (*n* = 7/16).

**Table 1:** Demographic and clinical data of patients with AAN and comparison of seropositive and seronegative patients (data are presented as the mean ± standard deviation)

	AAN cohort	Seropositive	Seronegative	Differences between s + ^2^/ s−^3^
	*n* = 17	*n* = 12	*n* = 5	
Age at first visit, y^1^	51.2 ± 6.8	50.5 ± 7.6	52.8 ± 4.6	**n.s.**^**4**^
Sex	7 males (41.2%) 10 females (58.8%)	5 males (41.7%) 7 females (58.3%)	2 males (40.0%) 3 females (60%)	**n.s**
Antibodies				
N-type Ca channel	8 (47.1%)	8 (66.7%)	0	
gACHR	3 (17.6%)	3 (25%)	0	
IENFD (*n* = 16), fibres/mm	5.74 ± 4.98	4.53 ± 4.91	8.4 ± 4.45	**n.s**
Autoimmune diseases	10 (58.8%)	7 (58.3%)^5^	3 (60%)^6^	n.s
Neoplasia	2 (11.8%)	1 (8.3%) (Thyroid carcinoma)	1 (20%) (breast cancer)	n.s

Bold indicates significance level was *p* > 0.05

^1^years, ^2^seropositive, ^3^seronegative, ^4^not significant, ^5^Hashimoto’s diseases *n* = 2, Ankylosing spondylitis, Sjögren’s disease, sarcoidosis, psoriasis and stiff limb syndrome: each *n* = 1, ^6^Hashimoto’s disease *n* = 2, Sjögren’s disease *n* = 1

All patients described gastrointestinal (17/17, 100%) and bladder dysfunction (17/17, 100%). Many patients had circulatory and orthostatic symptoms (16/17, 94.1%), complaints with regard to disturbed vision (blurred vision, glared perception, 10/15, 66.7%), dry skin (13/16, 81.3%) and pathological sweating (hypo/hyperhidrosis/sudomotor dysfunction, 10/14, 71.4%). A total of 82.4% (*n* = 14) suffered from at least one episode with sensory symptoms such as paraesthesia. Neuropathic pain was described in only 41.2% (*n* = 7).

**Fig. 2:**
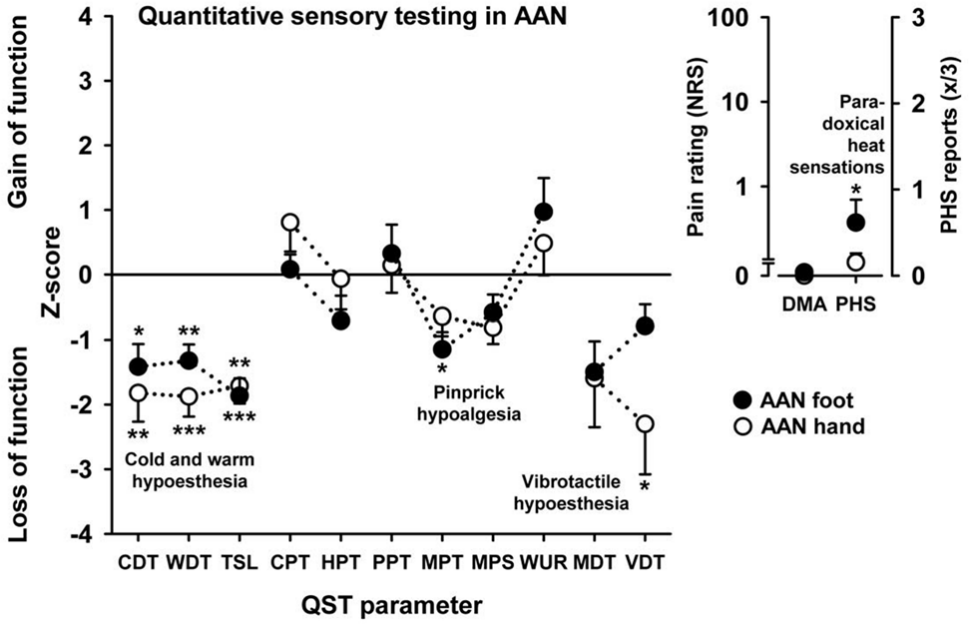
Loss and gain of function in the QST examination of AAN patients over the hand and feet. The QST *z* score profile shows a more generalized small- (CDT, WDT, TSL) rather than large-fibre (VDT) neuropathy for nonpainful thermal and mechanical stimuli than for painful mechanical stimuli (MPT). For abbreviations, see the legend of Table 2 and *DMA* dynamic mechanical allodynia, *PHS* paradoxical heat sensation. Stars denote the level of significance; *p < 0.05, **p < 0.01, ***p < 0.001. Figure was created using SigmaPlot (Version 11, Systat Software, Germany)

Examination of the z score QST data revealed significant loss of function for the performance of small fibre-mediated nonpainful thermal stimuli such as warm (*p* < 0.001) and cold detection (*p* < 0.01) over hands and feet (Table 2). Further deficits were found for the vibration detection threshold (VDT) over the hands using a Rydel-Seiffer tuning fork (*p* < 0.05). Moreover, the mechanical pain threshold (MPT) to pinprick stimuli was reduced over the feet (*p* < 0.05), and paradoxical heat sensation was increased over the feet (*p* < 0.05, *T* test). All QST parameters were comparably altered over the hands and feet with the exception of the VDT (hand vs. foot difference: *p* < 0.05) and MPT (hand vs. foot difference: *p* < 0.05), demonstrating a nonregional SFN (see Fig. 2). Subgroup analyses of seropositive vs. seronegative AAN patients did not show any significant differences with the exception of one of 26 QST parameters (MDT *p* < 0.05).

**Table 2:** Mixed two-way ANOVAs of QST data from AAN patients vs. controls (each N = 13) with a main effect pronounced for small- rather than for large-fibre function

QST parameter	Between factor 1 AAN vs. controls		Within factor 2 hand vs. foot	
	*F* value	*P* value	*F* value	*P* value
CDT^1^	13.61	< 0.01**	0.95	n.s
WDT^2^	30.54	< 0.001***	1.05	n.s
TSL^3^	27.84	< 0.001***	0.16	n.s
CPT^4^	1.65	n.s	1.74	n.s
HPT^5^	0.73	n.s	1.83	n.s
PPT^6^	0.25	n.s	0.16	n.s
MPT^7^	5.57	< 0.05*	4.51	< 0.05*
MPS^8^	3.07	0.09	0.4	n.s
WUR^9^	1.06	n.s	2.3	n.s
MDT^9^	5.65	< 0.05*	0.05	n.s
VDT^10^	6.95	< 0.05*	6.34	< 0.05*

This effect was observed for nonpainful stimuli rather than to a very slight extent for painful mechanical stimuli. The almost absent hand vs. foot differences indicate that the pronounced sensory deficits in AAN were nonregional. Stars denote the level of significance with

*Significant at a level of 0.05

**Significant at a level of 0.01

***Significant at a level of 0.001

^1^Cold detection threshold; ^2^warm detection threshold; ^3^thermal sensory limit; ^4^cold pain threshold; ^5^heat pain threshold; ^6^pressure pain threshold; ^7^mechanical pain threshold; ^8^mechanical pain sensitivity; ^9^wind up ratio, ^9^mechanical detection threshold; ^10^vibration detection threshold;

Skin biopsies revealed reduced IENFD in 10 of 17 patients (58.8%). There was no significant difference between seronegative vs. seropositive patients concerning IENFD (*p* > 0.05). There were no meaningful correlations between QST values and the IENFD because of large scattering. No significant differences concerning autonomic parameters were found between patients with and without nerve fibre damage.

## Discussion

It is a new and important finding that based on QST and skin biopsies, most AAN patients in this study had signs of a nonregional SFN regardless of autoantibody serostatus. The nonregional distribution across hands and feet is suggestive of a generalized neuropathy rather than a length-dependent peripheral neuropathy.

QST examination revealed a significant loss of function for the perception of warmth and cold as well as deficits in the perception of non-noxious vibrotactile sensation. Additionally, a significantly altered MPT was found. With the exception of the A-beta fibre-mediated vibratory detection threshold, all other stimuli are mediated by thinly myelinated A-delta (cold, pinprick) or unmyelinated C fibres (warm), compatible with a small fibre rather than a large-fibre neuropathy in AAN patients. This effect was observed for nonpainful stimuli rather than for painful mechanical stimuli (Table 2). No significant differences in sensory deficits were found between hands and feet except for VDT and MPT, indicating nonregional and possibly generalized sensory deficits in AAN. However, the slightly significant deficits for MPT and VDT should not be overinterpreted, as the analysis was not controlled for multiple comparisons. Interestingly, a previous retrospective study on 6 AAN patients also reported reduced and diffusely spread pinprick hypoalgesia in one case [3].

In accordance with previous results, most patients were seropositive [1, 4]. Compared to published data, we found a rather low incidence of gACHR [1] but a higher incidence of N-type calcium channel antibodies [4]. Variations in the occurrence of antibodies may have been caused by a smaller number of tests for N-type calcium channel antibodies, or they may have been a random effect because of the overall small number of subjects.

Subgroup analyses for antibody-positive vs. antibody-negative patients did not show any significant group differences except for one of the 26 QST parameters (MDT). These incidental findings should be interpreted with caution because this analysis was not corrected for multiple comparisons and the deviation is within the range of false positive results to be expected. It may therefore be assumed that seropositive patients may differ regarding other clinical features [3] but not for sensory dysfunction and the presence of small/large fibre neuropathy. This hypothesis is confirmed by the analysis of skin biopsies that also did not show any significant differences between seropositive and seronegative patients (Table 3).

**Table 3:** Comparison of autonomic complaints between AAN patients with small fibre damage in the skin biopsy (*n* = 10) and normal intraepithelial nerve fibre density (*n* = 7)

	AAN cohort	AAN + small fibre damage in skin biopsy	AAN with normal intraepithelial nerve fibre density	Differences between groups
Hypo-/Anhidrosis/heat intolerance	71.4% (*n* = 10/14)	75% (n = 6/8)	66.7% (*n* = 4)	n.s
Disturbed vision	66.7% (*n* = 10/15)	55.6% (*n* = 5/9)	83.3% (*n* = 5/6)	n.s
Circulatory disorder	94.1% (*n* = 16/17)	90% (*n* = 9/10)	100% (*n* = 7/7)	n.s
Bladder disorder	100% (*n* = 17/17)	100% (*n* = 10/10)	100% (*n* = 7/7)	n.s
Dry skin	81.3% (*n* = 13/16)	88.9% (*n* = 8/9)	71.4% (*n* = 5/7)	n.s
Gastrointestinal symptoms	100% (*n* = 17/17)	100% (*n* = 10/10)	100% (*n* = 7/7)	n.s
OH in tilt table	58.8% (*n* = 10/17)	70% (*n* = 7/10)	42.9% (*n* = 3/7)	n.s
Norepinephrine level supine^1^	317.3(± 186.1)	266 (± 186.2)	385.7 (± 178.1)	n.s
Norepinephrine level upright^1^	541.1(± 515.7)	500 (± 583.9)	613 (± 440.3)	n.s

Data are presented as the mean ± standard deviation

^1^pg/ml

SFN occurs in a general population with a prevalence of 53 patients per 100,000 [14]. Our results indicate a high prevalence of signs of SFN in AAN patients. Approximately 59% of patients showed abnormalities in skin biopsies, such as reduced IENFD. Clinical signs were sensory deficits, such as dysesthesia in at least 82.4% of patients, and QST values also showed small- rather than large-fibre dysfunction compared to the state of healthy controls. SFN reflects damage to the small unmyelinated and thinly myelinated nerve fibres [5] that are also affected in AAN. The common occurrence of SFN in AAN and the damage of the affected nerve fibres is compatible with the hypothesis that SFN might be one possible cause of AAN. On the other hand, autoimmune factors causing the AAN itself might also be one of the causes of SFN. This open question is still under discussion. For example a “preliminary evidence of dysimmune causes” [15] for these neuropathies is described by Oaklander. Others [16] also mentioned AAN as a secondary cause of SFN in their review*.* Nonetheless, the clinical presentation of SFN in AAN patients does not match the classic distal symmetric presentation of “idiopathic” SFN. AAN is defined by severe autonomic failure, which is, to this extent, not typical for classical SFN. Sensory symptoms of SFN, such as paraesthesia [5], were also evident in the AAN patients in the present study. In contrast, neuropathic pain, another major characteristic feature of idiopathic SFN [5], occurred in only approximately 40% of our patients. There are also differences in QST examination and distribution of sensory deficits. Typical SFN presents itself in a length-dependent form; many fewer patients show a non-length-dependent form [17]. In the non-length-dependent form, younger women with autoimmune diseases are more frequently reported [17]. The generalized distribution of small fibre impairment in AAN patients and the patients’ characteristics (female > male, autoimmune cause) are compatible with the latter, non-length-dependent type of SFN. Similar results were found in women with postural tachycardia syndrome [18]. AAN patients with and without nerve fibre damage seemed to be equally affected by autonomic symptoms, but these results have to be interpreted with caution because of the small sample size.

However, even though limitations exist due the retrospective character of the present study, small cohort size and limited homogeneity of the patient population, the initial results are promising.

Nevertheless, larger prospective studies are needed to confirm the association between SFN and AAN. Such studies might also include skin biopsies not only from the distal but also from the proximal leg, providing further information about the distribution of SFN in AAN patients. For clinical practice, we would propose screening patients with autonomic neuropathies for symptoms of small fibre nerve damage, or abnormalities in physical examinations that are indicative of a small fibre damage. Dependent on these results, clinicians should consider securing the diagnosis via QST and skin biopsy, if available. A definite diagnosis of SFN might also have implications for further genetic diagnostics (e.g. treatable Fabry disease or transthyretin amyloidosis in patients with SFN in former suspected AAN) and possible immune therapy in autoimmune cases.”

The set of inclusion criteria was based on the current literature and clinical experience acquired over years diagnosing and treating patients with AAN in our autonomic nervous system centre. Due to the strict inclusion and exclusion criteria, the study only included patients with the typical presentation of AAN. Thus, further studies may investigate whether the results can be generalized to even younger or older patients with possible AAN or patients with an atypical presentation, such as gastrointestinal pseudo-obstruction.

In conclusion, QST and skin biopsies revealed a small fibre degeneration in AAN. This was a nonregional finding according to the QST results, without relevant differences between seropositive and seronegative patients. Antibodies against the N-type calcium channel were more common than gACHR-Ab. The presentation of SFN in AAN patients is different from that of typical SFN. In AAN patients, autonomic complaints rather than neuropathic pain are predominant. Autoimmunity in AAN patients might damage small fibres, but not all of the AAN patients developed a SFN*.* Further studies are needed to better understand the typical presentation, pathophysiology, course and treatment of SFN in AAN.

## Data Availability

Data are available on request from the first authors.
